# Proton Conductivity of Nafion/*Ex-Situ* Sulfonic Acid-Modified Stöber Silica Nanocomposite Membranes As a Function of Temperature, Silica Particles Size and Surface Modification

**DOI:** 10.3390/membranes6010012

**Published:** 2016-01-28

**Authors:** Beatrice Muriithi, Douglas A. Loy

**Affiliations:** 1 Waters Corporation, Milford, MA 01757, USA; Beatrice_Muriithi@waters.com; 2 Department of Materials Science and Engineering, University of Arizona, Tucson, AZ 85721, USA; 3 Department of Chemistry and Biochemistry, University of Arizona, Tucson, AZ 85721, USA

**Keywords:** Stöber silica particles, water uptake, Proton conductivity, Nafion/*ex-situ* silica nanocomposite membrane, sulfonic acid-modified silica particles

## Abstract

The introduction of sulfonic acid modified silica in Nafion nanocomposite membranes is a good method of improving the Nafion performance at high temperature and low relative humidity. Sulfonic acid-modified silica is bifunctional, with silica phase expected to offer an improvement in membranes hydration while sulfonic groups enhance proton conductivity. However, as discussed in this paper, this may not always be the case. Proton conductivity enhancement of Nafion nanocomposite membranes is very dependent on silica particle size, sometimes depending on experimental conditions, and by surface modification. In this study, Sulfonated Preconcentrated Nafion Stober Silica composites (SPNSS) were prepared by modification of Stober silica particles with mercaptopropyltriethoxysilane, dispersing the particles into a preconcentrated solution of Nafion, then casting the membranes. The mercapto groups were oxidized to sulfonic acids by heating the membranes in 10 wt % hydrogen peroxide for 1 h. At 80 °C and 100% relative humidity, a 20%–30% enhancement of proton conductivity was only observed when sulfonic acid modified particle less than 50 nm in diameter were used. At 120 °C, and 100% humidity, proton conductivity increased by 22%–42% with sulfonated particles with small particles showing the greatest enhancement. At 120 °C and 50% humidity, the sulfonated particles are less efficient at keeping the membranes hydrated, and the composites underperform Nafion and silica-Nafion nanocomposite membranes.

## 1. Introduction

Proton conductivity of Nafion, still considered the benchmark for polymer electrolyte membranes in PEM fuel cells, drops with temperatures >100 °C, limiting full exploitation of advantages of operating PEM fuel cells at temperatures above the boiling point of water [1,2,3]. The drop in performance has been attributed to dehydration and microstructural changes that close down conducting channels [1]. Dehydration of Nafion can be reduced with the incorporation of hygroscopic oxides [3], the efficacy of which has been shown to be particle size dependent [4,5]. While hydration is improved and membrane failure is reduced at elevated temperatures, proton conductivity is often lower with metal oxide modified Nafion membranes due to volumetric dilution of sulfonic groups in membranes [6,7,8]. This can be minimized by modifying the silica particles with sulfonic acid groups [9,10,11].

**Scheme 1:**
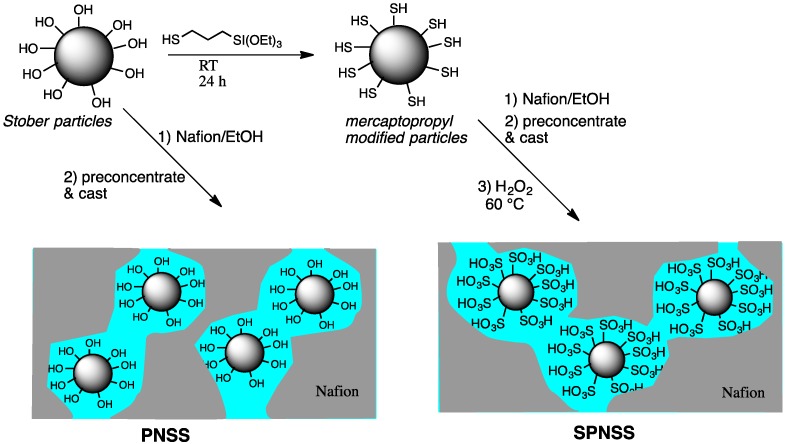
Preparation of nanocomposite membranes of silica (PNSS) or sulfonic acid modified (SPNSS) silica with Nafion.

While there are a number of different approaches to attaching sulfonic acid groups on silica particles, the most common is through formation of hybrid particles based on mercaptopropyltrialkoxyilanes and tetraethoxyilane followed by oxidation of the mercapto group (SH) to the sulfonic acid. The sulfonated particles can be made in the membrane by soaking the Nafion with precursors [12,13,14], or outside the membrane by silating silica particles with the mercaptopropyltriethoxysilane [15,16,17]. For fuel cell membranes, the final oxidation is carried out in the final composite membrane during the hydrogen peroxide oxidation treatment of the Nafion membrane [13,18,19,20]. To date, no one has examined the influence of the size of the sulfonated silica particles on the performance of the Nafion composite membranes. Previous studies of Nafion composites with silica particles without surface modification did show that composites with smaller silica particles exhibited superior ionic conductivity and water retention at higher temperatures [4,5,21]. In this study, our goal was to extend the particle size study of Nafion nanocomposites with surface sulfonated silica to prevent reductions in proton conductivity due to dilution of sulfonic acid groups by silica and to ascertain the influences of the particle size on water uptake, ionic exchange capacity, and proton conductivity. Silica particles were prepared by a simple, modified Stöber procedure to have narrow particle size distributions with average diameters ranging from 20–235 nm [4,22]. Particles were surface modified with 3-mercaptopropyltriethoxysilane. Sulfonated Preconcentrated Nafion Stöber Silica (SPNSS) nanocomposite membranes were prepared by dispersal of the particles in ethanol solutions of Nafion, preconcentrated by evaporation to a viscosity high enough to prevent particle floatation, and casting (Scheme 1). Finally, the mercapto groups were oxidized in the membranes to sulfonic acids with hydrogen peroxide. The resulting SPNSS membranes were compared with Nafion 117 and membranes prepared with un-modified silica (Preconcentrated Nafion Stöber Silica or PNSS nanocomposite membranes).

## 2. Results and Discussion

### 2.1. Nanoparticle Growth, Size, and Surface Modification

To look at the influence of particle size on the nanocomposite membrane properties, it is necessary to prepare spherical silica particles with control over the size and a narrow size distribution. One of the most commonly used approaches for monomodal silica particles of less than 5 microns in diameter is using a Stöber procedure. Using a modified version of this method, silica particles (10–300 nm) were prepared by changing concentration of ammonium hydroxide while keeping other reagents (TEOS and anhydrous ethanol) constant [5]. Particle size and distributions were determined using dynamic light scattering and confirmed with atomic force and scanning electron microscopies. Modification of particles was performed by adding 3 wt % mercaptopropyltriethoxysilane to the Stöber polymerization after 24 h. After 12 h, the particles were isolated by centrifugation and characterized.

For the measurement of particle IECs, the mercapto groups on the surface modified particles were oxidized with hydrogen peroxide without incorporation in Nafion. Titration of the unmodified silica particles revealed greater numbers of silanols than expected for non-porous silica (Table 1) collaborating nitrogen sorption surface area measurements that the particles were porous. Increase in IEC with the size of the unmodified particles can be attributed to more porosity in the larger Stober particles [4]. Surface modification of the silica particles with sulfonic acid groups increased the IEC of the smaller particles more than the larger particles suggesting that the geometric surface area, not the interior pores, were silated [23].

**Table 1:** The impact of surface modification on the Ion Exchange Capacities (IECs) of Stöber silica nanoparticles. Particle size was measured by dynamic light scattering (DLS) and IECs were determined by titration.

Particles with Silanols (PNSS)			Sulfonic Acid Modified Particles (SPNSS)		
Particle Size (nm)	Titrated SiOH (mmol/gram)	Theoretical Surface * SiOH (mmol/gram)	Particle Size (nm)	Titrated Acidic Groups (mmol/gram)	Theoretical Surface SiOH (mmol/gram)
50 ± 2	1.48	0.38	40 ± 5	3.86	0.47
120 ± 8	1.18	0.16	80 ± 8	2.24	0.24
232 ± 16	1.62	0.08	140 ± 10	2.05	0.13
388 ± 10	1.78	0.05	232 ± 16	1.8	0.08

* calculated geometrically based on experimentally determined size and a density of 2.26 g·cm^−3^.

### 2.2. Impact of Stöber Silica Particle Surface Modification on Nafion/ex-situ Sulfonated Silica Nanocomposite Membranes (SPNSS) Ionic Exchange Capacity

Nanocomposite membranes were prepared by dispersing 5 wt % of the silica particles (relative to the mass of Nafion) into a solution of Nafion in ethanol. The viscosity of the dispersion was increased to 65 centipoise by evaporation of ethanol, whereupon 90 micron membranes were prepared by casting in petri dishes. Once dry the transparent membranes were steeped in 10% H_2_O_2_ for 1 h at 60 °C to oxidize the mercpato groups to sulfonic acid groups and to prepare the Nafion nanocomposite membrane for testing. Examination of cross-sections of the membranes by scanning electron microscopy revealed that the particles were dispersed homogeneously through the composite structure. Determination of IECs of the membranes provided a quantitative assessment of the amounts of acidic functionalities that were accessible as a function of particle size (Table 2). Nafion with a theoretical IEC of 0.91 mmoles SO_3_H groups per gram of polymer has sufficient acidic groups for its percolating network proton conducting channels. The first column of theoretical IECs for the PNSS and SPNSS membranes are based on the assumption that the silica particles are not acidic. The second column of theoretical IECs are based on the assumption that the acidic silanols or sulfonic acids on the silica particles participate in the ion exchange measurement. The agreement between the experimental data and the theoretical IECs based on non-acidic filler indicates that the acidic groups on either the un-modified silica in PNSS and the modified silica in the SPNSS materials are not adding to the ion exchange capacities. IECs of PNSS nanocomposite membranes (0.81) decrease with the addition of particles as Nafion sulfonic acid groups get replaced with less acidic silica phase, and also remained constant with respect to particle size [4,5]. However, when sulfonic acid functionalized particles are used, SPNSS nanocomposite membranes IECs (0.88–0.89) were very close to recast Nafion (0.9) due to some contribution from the sulfonic groups on the modified silica. The relative differences in IECs between the PNSS and SPNSS membranes are in agreement with differences in acidity between the constituent particles shown in Table 1. An increase in nanocomposite membranes’ acidity with functionalized silica has also been observed with *in-situ* generated functionalized silica in Nafion solutions or membranes [11,24].

**Table 2:** Comparison of ionic exchange capacity of SPNSS, PNSS, and Nafion membranes.

Membranes	Experimental IECs	Theoretical IECs with Non Acidic Filler	Theoretical IECs with Acidic Filler from Table 1
Nafion 117	0.93	0.91 (no filler)	0.91(no filler)
Recast Nafion	0.90 ± 0.03	0.91 (no filler)	0.91(no filler)
PNSS Membranes	0.81 ± 0.06	0.874	0.94
SPNSS-30nm Membranes	0.88 ± 0.07	0.874	1.06
SPNSS-150nm Membranes	0.89 ± 0.2	0.874	0.97
SPNSS-235nm Membranes	0.80 ± 0.22	0.874	0.95

### 2.3. Water Uptake of SPNSS Nanocomposite Membranes at 60 °C

**Figure 1:**
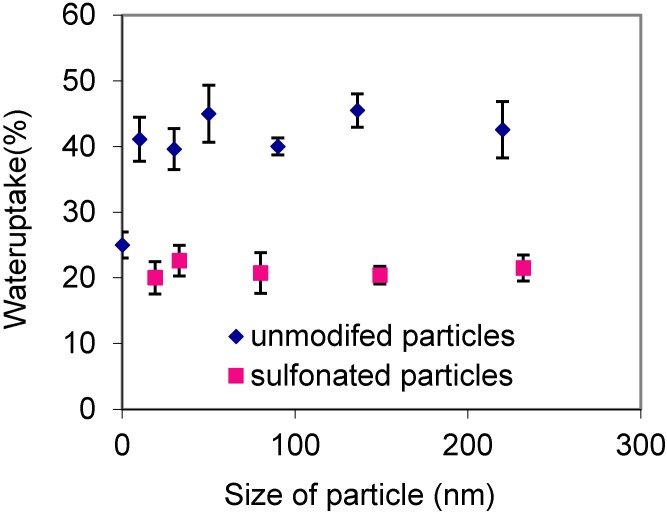
Water uptake for PNSS (blue diamonds) and SPNSS (red squares) membranes as a function of particle size.

Water uptake is performed by weighing the dry membranes, soaking them for X h in water at 60 °C, then reweighing the membranes. Nafion membranes from ethanol showed 25% water uptake by weight. The addition of 5 wt %silica nanoparticles in the Nafion increased the uptake to over 40%. Furthermore, we found that water uptake did not change as a function of particle size. It was out expectation that silica with sulfonic acid groups would have even greater water uptake. However, we discovered that the water uptake for the nanocomposite membranes with sulfonated silica nanoparticles was slightly lower than that of the recast Nafion membranes (Figure 1).

### 2.4. Proton Conductivity of SPNSS Nanocomposite Membranes at 80 °C

**Figure 2:**
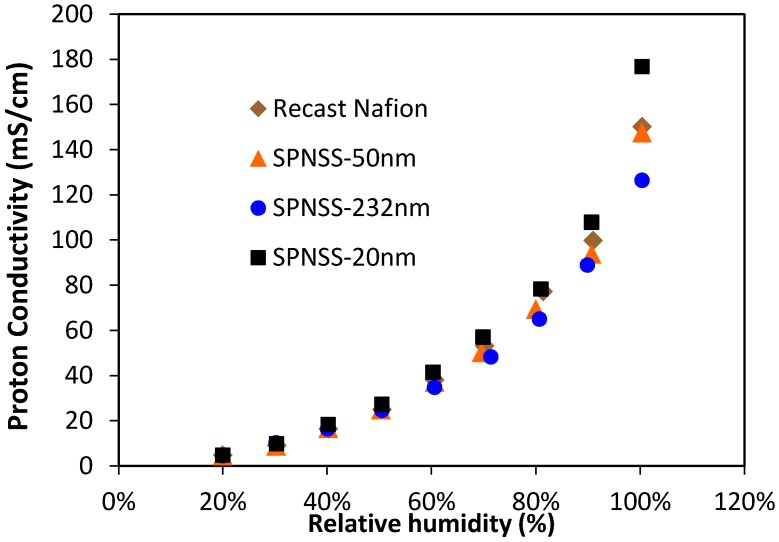
Proton conductivity of SPNSS nanocomposite membranes with recast Nafion at 80 °C. Proton conductivity increases with increase in relative humidity (RH) for all samples.

**Figure 3:**
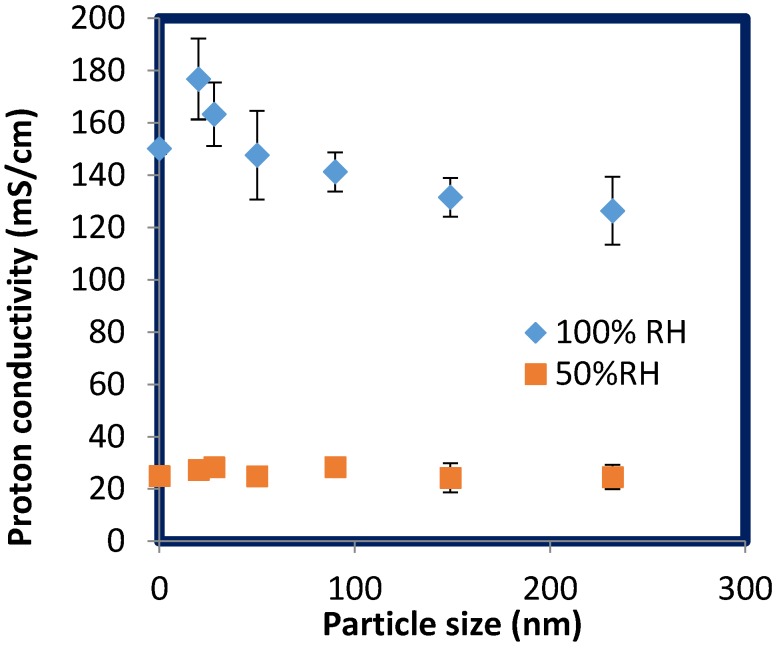
Impact of silica particle size on proton conductivity of SPNSS nanocomposite membranes for both 50% RH and 100% RH at 80 °C. Data at zero particle size corresponds to the data for recast Nafion.

Figure 2 shows proton conductivity of recast Nafion and SPNSS nanocomposite membranes as a function of relative humidity (RH) at 80 °C. Proton conductivity increasing with higher RH is attributed to the presence of different proton conduction mechanisms at various RH [5] suggesting that addition of sulfonated silica does not change these mechanisms. Proton conductivity of SPNSS nanocomposite membranes was higher than recast Nafion membranes with particles less than 50 nm. Proton conductivity of SPNSS nanocomposite membranes decreased with particle size at high RH as shown, and remained unchanged with particle size under lower humidity in Figure 3.

**Figure 4:**
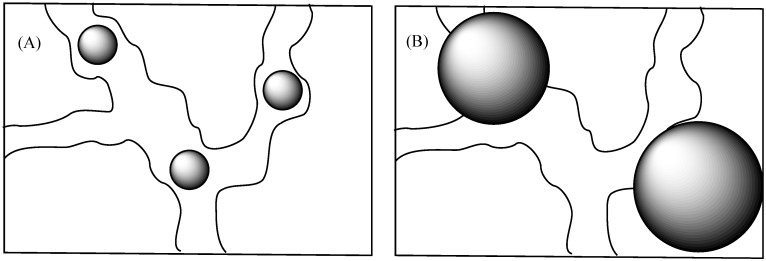
Influence of particle size on water channels in Nafion.

**Figure 5:**
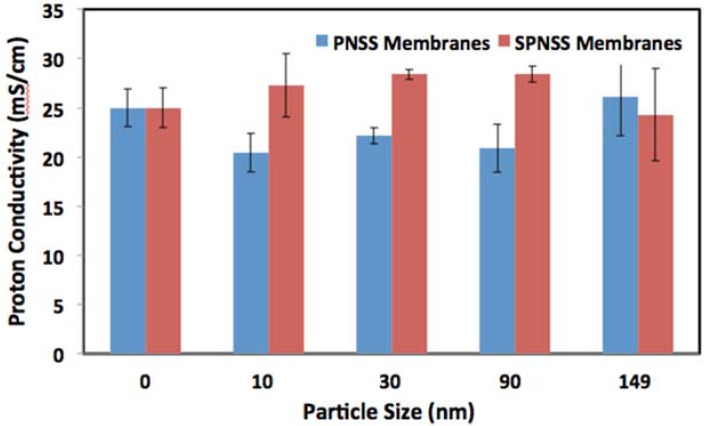
Comparison of the proton conductivity of PNSS and SPNSS nanocomposite membranes at 80 °C and 50% relative humidity.

**Figure 6:**
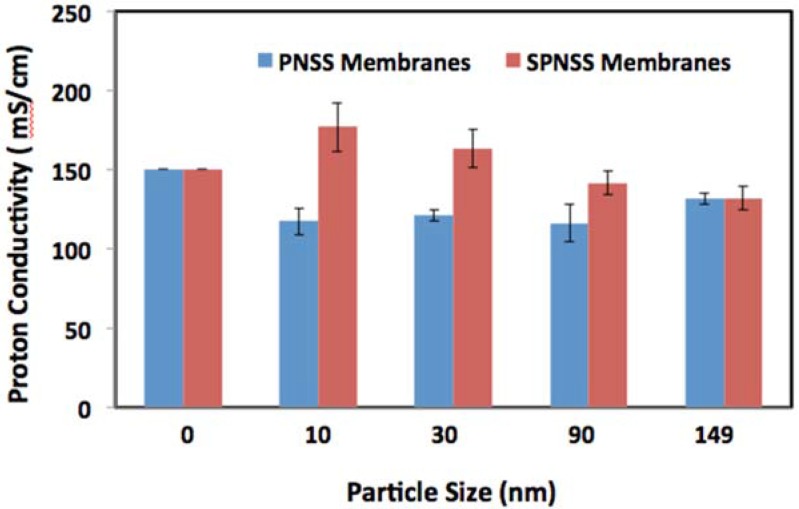
Comparison of the proton conductivity of SPNSS and PNSS nanocomposite membranes at 80 °C and 100% relative humidity.

At 80 °C and high RH, recast Nafion has higher proton conductivity compared to SPNSS nanocomposite membranes with particles >100nm. Under these conditions, Nafion has higher acidity than the SPNSS membranes with 150 and 232 nm particles and is highly hydrated. The presence of large particles in SPNSS nanocomposite membranes may block or interfere with the formation of the interconnected ionic channels in Nafion microstructures resulting into a reduction in proton conductivity (Figure 4). In this graph, the proton conductivity of the SPNSS nanocomposite membranes at low RH is more similar to that of recast Nafion. A closer look will show that the SPNSS membranes with smaller, modified silica particles did exhibit a significant increase in proton conductivity (Figure 5 and Figure 6). In earlier studies, proton conductivity of nanocomposite membranes prepared with unmodified particles (PNSS) was about 20% lower than recast Nafion at low RH which indicates that silica alone may add to the water uptake [5] but does not aid in proton conductivity. The greater proton conductivity of SPNSS nanocomposite membranes at low RH compared to those membranes prepared with unmodified silica particles may be due to increased acidity from the sulfonic groups on the particles.

Enhancement in proton conductivity of SPNSS membranes at higher RH was consistent with IEC values in Table 1 with smaller silica particles (<50 nm). In addition, small particles should generate less blockage of the ionic domains in the membranes microstructure than the larger particles.

A closer look at the proton conductivities of PNSS and SPNSS nanocomposite membranes at 80 °C corroborates these findings (Figure 5 and Figure 6). The proton conductivity of SPNSS nanocomposite membranes with sulfonated silica particles at low RH with small particles (<100 nm) is higher than for PNSS nanocomposite membranes with un-modified silica particles. It is also evident that the SPNSS membranes with particles smaller than 100 nm exhibit a slight, but significant increase in proton conductivity compared with the recast Nafion. SPNSS nanocomposite membranes prepared with particles >150 nm exhibit proton conductivity well below recast Nafion.

Larger silica particles likely increase proton pathway tortuosity and lower conductivity. The impact of particle size on proton conductivity has not been observed previously because in most prior cases sulfonated silica membranes were prepared via *in-situ* polymerization of tetraalkoxysilanes instead of preformed particles, making it difficult to control their size [25].

At high RH, the same trend was observed. SPNSS nanocomposite membranes with small particles have higher proton conductivity than PNSS nanocomposite membranes with similar size, un-modified particles. Interestingly, SPNSS nanocomposite membranes proton conductivity starts to decrease with particles size greater than 30 nm, merges with that of PNSS nanocomposite membranes when 149 nm particles are used and is significantly less than recast Nafion. Both modified and unmodified nanocomposite membranes made with 149 nm particles, irrespective of surface modification, had proton conductivities about 14.5% lower than recast Nafion. As with the membranes at low RH, SPNSS nanocomposites with particles larger than 150 nm showed even poorer proton conducitivies.

At 80 °C and high RH, recast Nafion is only surpassed by the nanocomposite membranes with small, sulfonated particles (SPNSS-10 and SPNSS-30). Therefore, at high RH, the proton conductivity of Nafion is enhanced by small sulfonic group modified particles, similar in size to the ionic domains. Larger particles (both unmodified and modified) may introduce tortuosity in proton pathways causing a reduction in proton conductivity in nanocomposite membranes.

### 2.5. Proton Conductivity of SPNSS Nanocomposite Membranes at 120 °C

Proton conductivity of SPNSS nanocomposite membranes is still dependent on the RH proton conduction requires hydration even at high temperatures. The highest proton conductivity of SPNSS nanocomposite membranes was 232.7 mS·cm^−1^ at 100% RH with 20 nm particles, whereas recast Nafion was only 164.2 mS·m^−1^ at the same condition, a 42% enhancement. Even with largest particles (232 nm), proton conductivity of the SPNSS nanocomposite membranes is about 22% higher than recast Nafion at 100% RH. SPNSS nanocomposites membranes with small particles (20 nm) have 16.4% higher proton conductivity than with large particles (232 nm). This increase in proton conductivity of nanocomposite membrane was consistent with particle acidity values and IEC values as shown in Table 1 and Table 2. SPNSS nanocomposite membranes did not perform as well at low RH, which may be attributed to their lower wateruptake [4].

**Figure 7:**
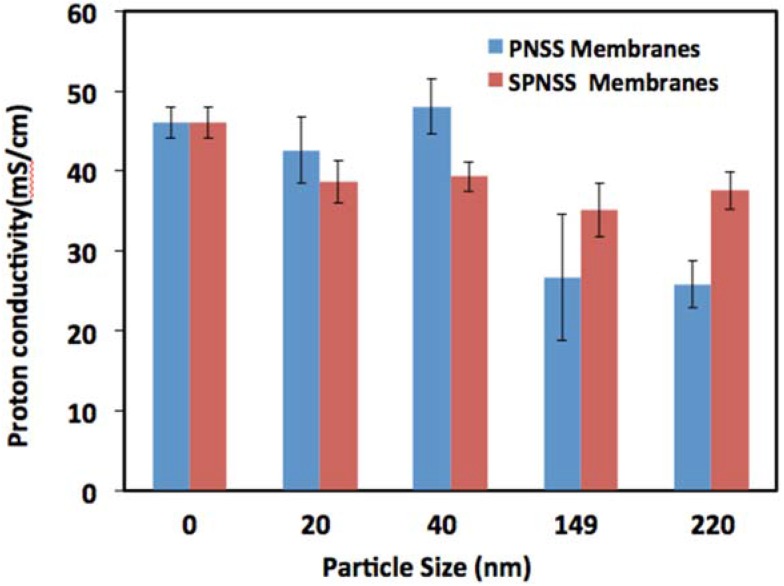
Comparison of proton conductivity of PNSS and SPNSS nanocomposite membranes at 120 °C and 50% relative humidity.

Figure 7 compares the proton conductivity of SPNSS and PNSS nanocomposite membranes at low RH (50%). Proton conductivity of PNSS nanocomposite membranes with small particles (< 50 nm) was higher (9%–18%) than with small, modified particles. The proton conductivity enhancement with the small unmodified particle was opposite of what was observed at 80 °C. PNSS nanocomposites’ membranes have higher water uptakes than SPNSS nanocomposites’ membranes, as reported previously [4]. The observed increase in proton conductivity with unmodified particles is indicative of the importance of membranes ability to retain water at high temperature and low RH over membranes acidity or IEC values. Adding smaller unmodified particles in Nafion increases the membranes’ ability to retain more accessible water at higher temperature resulting in enhanced proton conductivity at low RH [5].

Interestingly, at low RH (50%), the proton conductivity of SPNSS nanocomposites membranes with larger modified particles (>50 nm) is higher (24%–31%) than observed with PNSS nanocomposite membranes with similar particle size. Larger (>100 nm) silica particles may introduce some tortuosity in proton conducting channels, leading to lower proton conductivity. Proton conductivity of SPNSS nanocomposite membranes with large modified particles is enhanced due to the presence of sulfonic groups on the particles, which offer extra proton transport pathway and thus increasing proton conduction pathway connectivity. However, the performance of these SPNSS membranes prepared with larger particles (>50 nm) was still inferior to Nafion due to their lower ability to maintain hydration indicated by their lower water uptake.[5].

**Figure 8:**
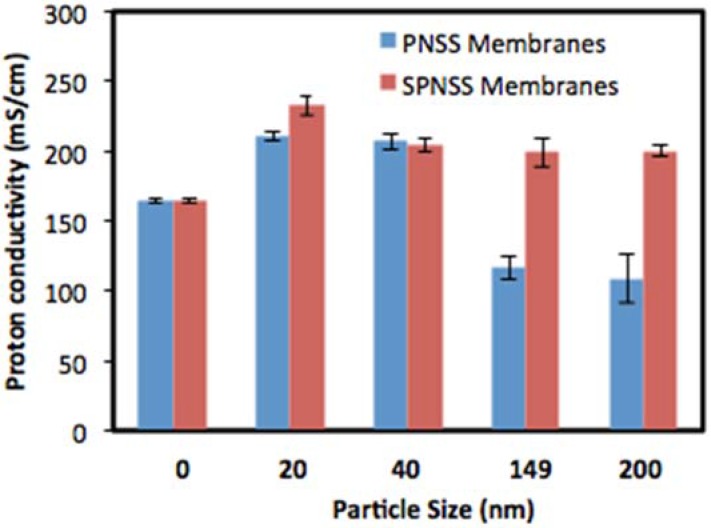
Comparison of proton conductivity of PNSS and SPNSS nanocomposite membranes at 120°C and 100% relative humidity.

At high RH, proton conductivity of SPNSS nanocomposite membranes is higher than recast Nafion irrespective of particle size (Figure 8). All of the SPNSS nanocomposite membranes are either higher or equal to PNSS nanocomposite membranes with similar particle size. Maximum proton conductivity of SPNSS nanocomposite membranes was 232.7 mS·cm^−1^ at 100% RH for membranes with 20 nm particles. Proton conductivity of SPNSS nanocomposite membranes with the largest particles (220 nm) is about 200 mS·cm^−1^ at 100% RH which is 83% enhancement compared with PNSS membranes and 30% higher than recast Nafion under similar conditions. The high proton conductivity observed with SPNSS membranes suggests that at high RH, the concentration of sulfonic acid groups in the membranes measured by the IEC values has a greater impact than the ability of membranes to retain more water. SPNSS nanocomposite membranes uptake 50% less water than PNSS nanocomposite membranes and about 20% less than recast Nafion. The increase in available sulfonic acid groups result in possible increase proton pathways and proton pathways percolation in SPNSS nanocomposite membranes thus enhanced proton conductivity. Previously, an increase in proton conductivity was observed with the addition of 171 nm sulfonated hollow silica particles at temperatures above 100 °C [17] which is in agreement with our findings.

At high RH, proton conductivity is dominated by the proton hopping mechanism [26,27]. Since there is enough membrane water, enahancement of proton conduction observed with the SPNSS nanocomposite membranes probably comes from increased concentration of sulfonic acid groups in membranes, making it easy for protons to hop from one sulfonic group to the next. In contrast, at low RH, the proton conductivity is dominated by the vehicle process (water assisted proton diffusion) and depends strongly on available water in the membranes [28].

The difference in proton conductivity between SPNSS membranes and PNSS membranes at low RH increases with increase in particle size due to the ability of small particles to hold more water (accessible) than large particles at high temperature. Under these conditions, the presence of water is more significant than the concentration of sulfonic acid groups. At higher RH, availability and connectivity of proton conduction pathways become more significant and the effect is reversed. SPNSS nanocomposite membranes with larger particles have enhanced proton conductivity compared to PNSS nanocomposite membranes with similar particle size due to the presence of extra sulfonic groups that offer improved proton conductivity pathways.

## 3. Experimental Section

### 3.1. General Methods

Nafion (5 wt %) in aliphatic alcohol, tetraethoxysilane (TEOS), concentrated ammonium hydroxide and anhydrous ethanol were obtained from Sigma Aldrich (St. Louis, MO, USA). Aqueous ammonia hydroxide was titrated with 0.1 N HCl with methyl orange as an indicator to determine its actual concentration (9M). TEOS was distilled from calcium hydride prior to use.

### 3.2. Stöber Synthesis

Silica nanoparticles were prepared by hydrolysis and condensation reactions of tetraethoxysilane in the presence of ammonium hydroxide according to a modified Stöber process [4,22]. For preparation of 30 nm silica nanoparticles, 14 mL of anhydrous ethanol and 0.5 mL of (9 M) ammonium hydroxide were added to a scintillation vial. The scintillation vials were tightly capped, and the solution was magnetically stirred for 5 min at 600 rpm, followed by addition of 0.5 mL of TEOS as quickly as possible (to avoid particle polydispersity) with continued stirring for 24 h. Control over particle size was achieved by changing the concentration of ammonium hydroxide as described previously [4]. Surface modification was achieved by adding about 3 wt % of 3-mercaptopropyltrimethoxysilane in the particle suspension and continued stirring for 12 h at 600 rpm and room temperature. After surface modification particles were washed with ethanol in three cycles of centrifugation and ultrasonic dispersion prior to analysis and use in the preparation of the Nafion/*ex-situ* sulfonated silica (SPNSS) nanocomposite membranes. Particles were characterized using dynamic light scattering (DLS), Atomic force microscopy (AFM) and scanning electron microscopy (SEM).

Particle surface modification was confirmedby diffuse IR, and quantification was carried out by titrations [29]. Samples for titration were prepared by drying washed particles in a vacuum oven at 120 °C for 12 h and weighed. Dry particles were dispersed in 20 mL of 0.05 M·NaOH (aq) at room temperature in scintillation vials. The vials were sealed and left stirring at 600 rpm for 12 h. Particles were centrifuged, and 5 mL of dispersant was collected and titrated with 0.05 M·HCL using phenolphthalein as an indicator. Silanol surface concentration (mmol/gram) was calculated using the equation below.

(1) Mmolgram=(VNaoH−VHCL)×CWdry

where V_NaOH_ is the volume of NaOH and V_HCl_ is the volume of HCl obtained from titration, W_dry_ is particles dry weight and C is concentration of NaOH, which equal concentrations of HCl, which is 0.05 M.

### 3.3. Nanocomposite Membranes

Nanocomposite membrane (PNSS & SPNSS) were prepared by mixing Nafion^®^ solution with enough Stöber silica nanoparticles (in ethanol) to make 5 wt % particles in the final Nafion^®^ membranes. Mixture was stirred for overnight, then placed in a conventional oven at 70 °C for 2 hresulting in a 50% reduction in volume and an increase in solution viscosity from 14.5 ± 1.2 to 64.6 ± 1.4 centipoises. The mixture was then stirred at 600 rpm for 4 h to re-disperse the particles in the polymer solution. Membranes were cast by pouring the viscous mixture in a glass petri dish (60 mm × 15 mm) at room temperature and the temperature raised gradually to 60 °C in the oven. The membranes were left to dry overnight at 60 °C, and finally annealed at 90 °C for 30 min. Once dry, the membranes were detached from the petri dish by soaking in deionized water for about 2 min. Nafion^®^ nanocomposite membranes with functionalized particles were prepared in a similar manner and are designated with the SPNSS acronym.

Membranes morphology and particle distribution were characterized using SEM and (AFM). SPNSS nanocomposites membranes were soaked in 10 wt % hydrogen peroxide at 60 °C for 1 h to oxidize the mercapto groups on the particles’ surfaces followed by rinsing with a copious amount of DI water. SPNSS nanocomposite membranes were soaked in 0.5 M sulfuric acid to acidify them and again rinsed with copious DI water. These pretreated membranes were soaked in DI water for 24 h prior to analysis and use.

### 3.4. Water Up-Take Measurements

Pretreated membranes were dried at 100 °C for 2 h in a vacuum oven and weighed (dry weight), then soaked in DI water at 60 °C for (high temperature) and 25 °C (low temperature) for 2 h, blotted dry and weighed (wet weight). Water-uptake values were calculated from the equation 2 below.

(2) $$wateruptake=\frac{W_{wet}-W_{dry}}{W_{dry}}\times 100$$

### 3.5. IEC Measurements

The ionic exchange capacity was determined for membranes which had been pretreated as described previously. Membranes were dried at 100 °C for 2 h in a vacuum oven to remove excess water. Dry membranes were weighed and then soaked in a 1M NaCl solution at room temperature overnight to exchange Na^+^ ions with H^+^ in the membranes. The Na-form membranes were removed from the solution, and the solution was titrated to end point with 0.1 M·NaOH solution. The endpoint of the titration was determined with both a pH probe and phenolphthalein as an indicator. The quantity of exchanged H^+^ ions was calculated from the volume of the NaOH used. Finally, the EW was calculated using the dry weight of the polymer and the quantity of exchanged protons using the Equation (3) below [19,20].

(3) $$\mathrm{EW}\left(\frac{g}{\mathrm{mol}}\right)=\frac{m}{\left[\mathrm{NaOH}\right]\times V_{\mathrm{NaOH}}}\times 1000$$

where m is the mass of the polymer and [NaOH] and V_NaOH_ are the concentration and volume of the NaOH used in the titration.

The ion exchange capacity is calculated from the EW following the following equation

(4) IEC(mmolg)=1000EW

### 3.6. Proton Conductivity Measurements

**Scheme 2:**
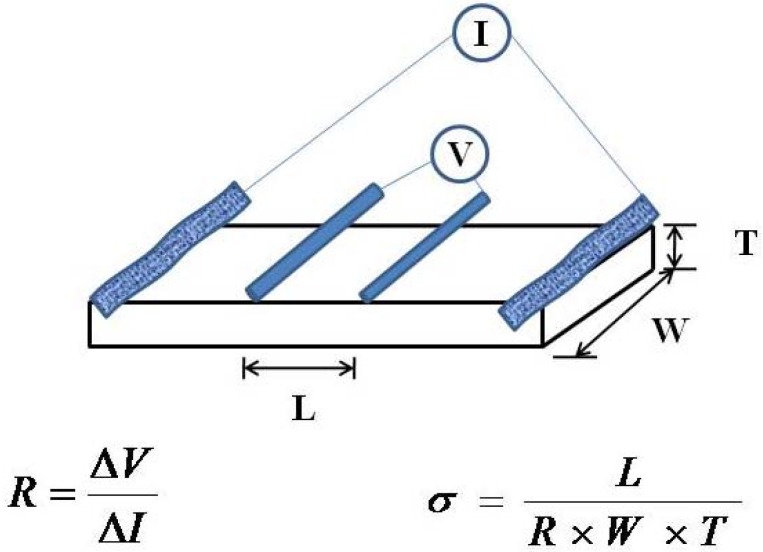
Configuration for measuring proton conductivities with equations used in calculations.

Proton conductivities of Nafion and Nafion nanocomposite membranes were measured by a DC method with a system developed by BeKKtech for membranes which had been pretreated as described previously. Pretreated membranes were soaked in DI water for 24 h prior the experiments. A rectangular strip of membrane (at least 2 cm in length, and 5 mm width) was placed in a four-probe Teflon cell (BekkTech, Loveland, CO, USA) that consisted of two outer platinum foils and two inner platinum electrodes. This was connected to the BekkTech test stand for the continuous relative humidity and temperature control equipped with aBT-514 gas delivery system (BekkTech, Loveland, CO, USA). The measurements were performed at 80 °C and 120 °C with relative humidity ranging from 20% to 100%. Measurements are carried out by applying a voltage (1 V) on the outside electrodes, and resistance to current flow in the inside electrodes is measured and the conductivity of the membranes was determined using the Scheme 2 shown below.

The data was processed using excel. At least three measurements were carried out for each nanocomposite membrane, and the average was determined.

### 3.7. Microscopy

Samples were analyzed by tapping mode AFM in air using the Dimension 3100 nanoscope (Veeco instruments, Santa Barbara, CA, USA) and silicon nitride tapping cantilever with force constant of 42 mN/m. The AFM instrument used for this work was calibrated with calibration grids from Veeco regularly. All AFM tips were ozone cleaned for 1 h prior use to remove organic contaminations. The images were processed using Nanoscope 5.12r5 software from Veeco instruments (Plainview, NY, USA). Samples were imaged several times on several spots on the same sample in order to check for sample homogeneity. Samples were prepared by mounting the membrane on a sample holder using carbon adhesive stabs. Morphology of the samples was analyzed using tapping mode AFM without additional sample preparation. However, samples for SEM analysis were coated with platinum at 6 mA and 7 V for 90 s prior the experiments. Images were obtained at an accelerating voltage of 2 KV and current 10 µA.

Scanning electron microscopy analysis of the nanocomposite membranes was conducted on a Hitachi S-4800 field-emission microscope (FE-SEM) with an accelerating voltage of 15 keV. The accelerating voltage was lowered between 10 and 5 keV when imaging nanocomposite membranes until a good image was observed then captured. All nanocomposite membranes were gold sputter-coated before analysis using a Hummer sputtering system. Coatings were applied for 60 s at 15 mA. This deposition leads to gold coatings that are approximately 4 nm thick.

## 4. Conclusions

Organic sulfonic acid (–SO_3_H) groups were successfully grafted on the surface of Stöber silica to improve proton conductivity of SPNSS nanocomposite membranes. The particles with modified with 3-mercaptopropylsilsesquixoxane groups and the mercapto were oxidized to the sulfonic acid groups. The performance of these nanocomposite membranes were evaluated on their proton conductivity at 80 °C and 120 °C and 0%–100% RH. This paper reports, for the first time, correlation between particle size, surface modification and nanocomposite membranes proton conductivity at different temperatures and RH. Proton conductivity enhancement of 20%–33%,was observed at 80 °C for all RH and particles and more significance with small particles (≤50 nm) about 33%. At this condition, it is significantly beneficial to use small sulfonic acid modified silica paricles to enhance the proton conductivity of Nafion. At high temperature (120 °C), several observations were made. The highest conductivity observed with SPNSS nanocomposite membranes was 232.7 mS·cm^−1^ at 100% RH and with 20 nm particles, whereas proton conductivity of PNSS nanocomposite membranes was only 210.4 mS·cm^−1^, 11% improvement under the same condition with same particle size. Proton conductivity of SPNSS nanocomposite membranes with the largest modified particles (220 nm) was 200 mS·cm^−1^ at 100% RH while PNSS membranes was only 109 mS·cm^−1^, 83% improvement which is much less than recast Nafion (164.2 mS·cm^−1^) under the same conditions. Greater benefits of using sulfonated particles are observed with large particles at high RH. In general, SPNSS nanocomposite membranes had significantly higher proton conductivity than PNSS membranes and recast Nafion at a higher temperature (120 °C) and high RH (100%).

At low RH, proton conductivity of SPNSS nanocomposite membranes with small particles (20 nm) was 38.6 mS·cm^−1^ and PNSS nanocomposite membranes was 42.6 mS·cm^−1^, a 10% decrease in performance was observed at same conditions with same particle size. With small particles, there are no benefits of using modified particles on proton conductivity at high temperatures. Proton conductivity of SPNSS nanocomposite membranes with large particle (220 nm) was 37.5 mS·cm^−1^ at 50% RH while with PNSS nanocomposite membranes was 25.8 mS·cm^−1^, 45% improvement in the same conditions and similar particle size. Proton conductivity of PNSS nanocomposite membranes was much lower than that of SPNSS nanocomposite membranes and even less than recast Nafion at 46.6 mS·cm^−1^. At 120 °C and low RH, no significance proton conductivity benefit is observed with particle-modified Nafion compared to recast Nafion.

These studies show the importance of particle size and explain why sometimes adding sulfonic groups on silica particles may not be beneficial depending on particle size and testing conditions such as temperature and relative humidity.
